# A review: Virulence factors of *Klebsiella pneumonia* as emerging infection on the food chain

**DOI:** 10.14202/vetworld.2022.2172-2179

**Published:** 2022-09-12

**Authors:** Katty Hendriana Priscilia Riwu, Mustofa Helmi Effendi, Fedik Abdul Rantam, Aswin Rafif Khairullah, Agus Widodo

**Affiliations:** 1 Doctoral Prgram in Veterinary Science, Faculty of Veterinary Medicine, Universitas Airlangga, Surabaya, East Java, Indonesia; 2 Department of Veterinary Public Health, Faculty of Veterinary Medicine, Universitas Airlangga, Surabaya, East Java, Indonesia; 3 Department of Veterinary Microbiology, Faculty of Veterinary Medicine, Universitas Airlangga, Surabaya, East Java, Indonesia

**Keywords:** biofilm, food chain, *Klebsiella pneumonia*, public health, virulence

## Abstract

Health problems can be caused by consuming foods that have been processed in unsanitary conditions; hence, the study of the impact of contamination on food and its prevention has become critical. The disease caused by *Klebsiella pneumoniae* in food is increasing significantly every year across the world. The main factors that are essential for the virulence of *K. pneumoniae* are lipopolysaccharide and polysaccharide capsules. Furthermore, *K*. *pneumoniae* is capable of forming biofilms. Capsule polysaccharides, fimbriae types 1 and 3, are crucial virulence factors contributing to biofilm formation in *K. pneumoniae*. The food contamination by *K. pneumoniae* may not directly pose a public health risk; however, the presence of *K. pneumoniae* refers to unhygienic practices in food handling. This article aims to demonstrate that *K. pneumoniae* should be considered as a potential pathogen that spreads through the food chain and that necessary precautions should be taken in the future.

## Introduction

*Klebsiella pneumoniae* predominantly has caused infectious diseases in immunosuppressed individuals. However, its emergence and spread are spreading even to healthy and immunocompromised people. Furthermore, the *K. pneumoniae* strain is becoming increasingly antibiotic-resistant, making treatment of infection with the strain extremely difficult [1]. The pathogenicity of *K. pneumoniae* bacteria is associated with several virulence factors that allow it to evade the host’s innate immune mechanisms. *Klebsiella pneumoniae* virulence factors include capsules, exopolysaccharides associated with mucoviscosity, lipopolysaccharides (LPSs), adhesins, and iron uptake systems. The factors that aggravate the infection caused by *K. pneumoniae* are multiple antibiotic resistance and its ability to cause nosocomial infections in humans. Furthermore, the previous case involved six Asian patients admitted to a US hospital with *K. pneumoniae* liver abscess; the gastrointestinal tract was suspected as the route of entry in one of the cases [2]. Moreover, *K. pneumoniae* causes infectious diseases such as pneumonia, meningitis, and blood and urinary tract infections [3]. *Klebsiella pneumoniae* is recognized by most physicians as the cause of community-acquired bacterial pneumonia. The opportunistic pathogen is the primary reason for the hospitalization of immunocompromised patients and individuals with severe diseases. *Klebsiella pneumoniae* is the main cause of nosocomial Klebsiella infection, a necrotic process that tends to attack the weak. In addition, *K. pneumoniae* can cause localized diseases and other related infections such as liver abscess, endophthalmitis, and meningitis in healthy individuals [4]. Apart from nosocomial infections, *K. pneumoniae* also spreads through contaminated food materials and is often considered an agent of foodborne illness. The pathogen can be found in seafood, frozen foods, and fresh meat [5]. In recent decades, foodborne outbreaks have highlighted the importance of developing and implementing preventive measures and programs to ensure food safety [6].

The observations of multidrug resistance (MDR) of *K. pneumoniae* from retail foods, and the presence of isolates associated with highly exposed sources of antibiotics, also make the possibility of antibiotic treatment difficult if the organism causes infection. The authors are concerned that this might be the case as our results are consistent with reports of antibiotic-resistant *K. pneumoniae* detected in various poultry and meat products [5].

Food hygiene and safety are based on various food safety issues, such as the presence of potential pathogens in food, toxins, resistance to antibiotics or sanitizers, and other virulence characteristics. The many benefits for consumers are in the globalization of trade because it produces a wider variety of high-quality foods that are easily available, affordable, safe, and meet the consumer’s needs. However, poor local infrastructure, characteristics of product sales, and lack of supervision in terms of sanitation are factors in the trade that can raise concerns about the potential for food poisoning due to microbiological contamination [7]. Consequently, it is prudent to exercise awareness of common and unexpected contaminants in food, such as *K. pneumoniae* and other antibiotic-resistant bacteria. In recent years, specifically in non-Asian cohorts, gastrointestinal transport has been recognized as a risk factor for *K. pneumoniae* colonization. Furthermore, in Australia, a recent study on the probability of transmission of *K. Pneumonia* revealed that 48% (13/27) of patients in the intensive care unit exhibited intestinal colonization before infection [8]. This review aimed to contemplate the potential risks of the pathogen in retail food hygiene, food safety, and public health and to investigate the virulence factors of *K. pneumoniae* as an emerging infection on the food chain.

## *Klebsiella pneumoniae* Virulence Factors and Pathogenicity

In addition to the clinical terrain, *K. pneumoniae* is present in foods, including raw vegetables, pulverized child formula, meat, fish, and road food [9]. In recent years, several foodborne outbreaks caused by *K. pneumoniae* have been reported in various countries. *Klebsiella pneumoniae* can express a variety of acridity factors, including capsule, endotoxin, siderophore, iron scavenging system, and adhesins, which play a crucial part in its pathogenesis. The capsule is a significant acridity factor, involved in at least two pathogenic mechanisms, that is, the protection of bacteria from phagocytosis and direct inhibition of the vulnerable host response. Several capsule types (K), especially, K1, K2, K54, K57, K20, and K5, are constantly associated with the pattern of community-acquired invasive pyogenic liver abscess, septicemia, and pneumonia. Moreover, K1, K2, K20, K54, and K57 are predominantly detrimental to experimental infections in mice and are frequently associated with severe infections in humans [10].

The capsules correspond to polysaccharides called K antigens, which are classified into 78 serological types. The capsule synthesis in *K. pneumoniae* is encoded by a gene located on the chromosomal operon, capsule polysaccharides (CPS). The CPS gene cluster hosts several genes i.e., *wzi*, *wza*, *wzb*, *wzc*, *gnd*, *wca*, *cpsB*, *cpsG*, and *galF*, that enable the formation of the capsule [11]. Furthermore, pathogenic bacteria require iron for their replication. Siderophores (iron carriers) are composites buried by microorganisms (similar to bacteria and fungi) to transport iron in the cell membranes. They have an advanced iron magnet than the host transport protein (transferrin). *Klebsiella pneumoniae* produce siderophores to gain iron from host iron-chelating proteins or the terrain for survival and reduplication during mammalian infection. The product of more than one siderophore by *K. pneumoniae* is a means to optimize the successful colonization of different napkins and/or avoid the neutralization of one siderophore by the host. Enterobactin, yersiniabactin, salmochelin, and aerobactin are different types of siderophores expressed by *K. pneumoniae*. The defining factors for high virulence and toxicity include capsules, siderophores, LPSs, and pili [12].

*Klebsiella pneumoniae* infection is caused by extended-spectrum beta-lactamase (ESBL)-producing bacteria and is resistant to carbapenems. ESBL can be caused by several beta-lactamases, encoded by genes such as TEM, SHV, CTX-M, and OXA [13]. The ESBLs have a greater impact on enhanced morbidity and mortality rates than bacteria that are not resistant to the infection. Nevertheless, multidrug-resistant or not, the capsule-deficient *K. pneumoniae* strains rarely result in complaints or complications in healthy individuals (except for urinary tract infections). In general, strains of *K. pneumoniae* or classic strains cause serious infections such as pneumonia, bacteremia, or even meningitis, when infecting individuals with compromised vulnerable systems, including diabetics [14].

The acridity of the *K. pneumoniae* strain was unaffected by transportation or the expression of medicine resistance, but it made treatment more difficult. Furthermore, due to its ability to infect both healthy and weakened vulnerable systems, a comparison of the *K. pneumoniae* hypermucoviscous (HV) strain and the classic *K. pneumoniae* (cKP) strain revealed that enterobactin expression is nearly ubiquitous among both strains and is thus considered to be the primary iron uptake system employed by *K. pneumoniae* [15]. Furthermore, the *irp* gene encodes the proteins required for yersiniabactin conflation, the *ybt* and *fyu* genes encode siderophores, and the *ybtQ* gene encodes their uptake receptors. However, *K. pneumoniae* has yet to fully characterize these. The strain is known as hypervirulent *K. pneumoniae* (hvKP), and it is more toxic, pathogenic, and causes a different cardiovascular disease than cKP [16]. Furthermore, in the absence of lipocalin-2, enterobactin promotes lung colonization and dispersion. In the presence of lipocalin-2; however, the strain of *K. pneumoniae* that produced only this siderophore was ruled out. Salmochelin is also the c-glucosylated form of enterobacterin. Iron mediates transport in iron-laden forms, and this modification prevents the list of salmochelin to lipocalin-2, preventing siderophore neutralization and lipocalin-2-dependent inflammation induction. Aerobactin is a siderophore composed of citrate and hydroxamate. Furthermore, the presence of aerobactin, which is occasionally expressed by clinical isolates of classical nosocomial *K. pneumoniae*, is always associated with hypercapsulation. However, not all hypercapsulated strains have aerobactin [17]. Furthermore, the single microorganism *K. pneumoniae* caused the liver abscess and is a concern as a new invasive syndrome [18]. It has been reported that *K. pneumoniae* has a 180 kbp plasmid containing the gene encoding aerobactin and its receptors, as well as the administration of *rmp*A, a mucoid phenotype. The comprehensive hvKp definition includes hypermucosal viscous phenotype, genotype, and clinical signs of metastatic infection [19].

Adhesins are cell surface factors or bacterial accessories that facilitate adhesion or attachment to other cells or shells, typically on the host where they infect or live. Adhesins are also a type of acridity factor. Adherence is a critical step in the pathogenesis of bacteria or infection that is required for the bacteria or infection to colonize a new host. Bacterial adhesions and adhesions are implicit targets for bacterial infection prevention or treatment. Fimbriae bonds (Fimbriae or pili) are structural proteins that help bacteria target specific kerchief shells in the host. There are three types of fimbriae in *K. pneumoniae*: 1, 3, and *K. pneumoniae* carbapenemase (KPC). Type 1 fimbriae are thin, hard, hair-like projections on the bacterial cell’s surface. Chaperones/intercellular pathways aggregate them, and the *fim* gene cluster decrypts them [20]. The *fimA* gene encodes the major structural subunit of *fimA*, while the *fimH* gene encodes the small-terminal adhesin subunit of *fimH*. Furthermore, the *fimK* gene is present in *K. pneumoniae* but not in *Escherichia coli*. The *fimK* gene is involved in the regulation of type 1 fimbriae, and it is important to note that its absence results in the failure of type 1 fimbriae expression [21]. In the urinary tract, the type 1 fimbriae gene is expressed, but not expressed in the gastrointestinal tract or lungs, so these fimbriae can foray into bladder cells and form a biofilm in the bladder. Furthermore, type 3 fimbria are spirally paraphrased by the *mrkABCD* gene cluster, which may be chromosomal or plasmid-deduced. The *fimH* and *mrkD* genes which render type 1 and type 3 fimbriae independently are responsible for attachment to host cells [22]. These factors are known to contribute to acridity and are responsible for colonization, irruption, and pathogenicity. Alarmingly, some studies reported that multi-drug-resistant, indeed carbapenem-resistant hvKP isolates have surfaced, which is a major public health concern [23, 24]. Furthermore, these are the main fimbriae that are important for biofilm conformation and for clinging to tissues and affinity in *K. pneumoniae*. The mrkA can bind to abiotic shells similar to medical bias before and after insertion into the patient’s body, whereas mrkD can bind to the extracellular matrix [25]. *Klebsiella pneumoniae*, particularly when hypermucoid, can cause invasive symptoms in a variety of species and is a common cause of mastitis in dairy farms. It can also thrive in a wide range of hosts and environmental niches, including water and soil. Several studies reported that despite having the *mrkD* and *fimH* genes encoding form 3 and type 1 fimbriae, respectively, hypermucoviscous isolates added serotype K1, demonstrating low upstream adhesion due to the presence of hypercapsule harboring these fimbriae [26].

Lipopolysaccharides and CPS are the two main factors responsible for the pathogenicity of microorganisms. Lipopolysaccharides contain antigens such as lipid A, core, and O-polysaccharide that are required for microorganisms to repel complement-mediated payoff. CPS is the pathogen’s most distant subcaste, and it is primarily involved in resistance to phagocytosis by polymorphonuclear cells by acting as a physical hedge [27]. As a result, both factors are necessary for microorganisms to spread through the blood and cause sepsis. However, little is known about how these two factors interact with *K. pneumoniae*. Due to active immunization with pure CPS-defended mice against the experimentally convinced *K. pneumoniae*, the experimental confirmation suggests that CPS may be important for *K. pneumoniae* conformation [28]. Similarly, monoclonal antibodies against *Klebsiella* CPS were found to reduce the inflexibility and hematogenic spread of *K. pneumoniae* in a recent study. Furthermore, CPS and LPS may play an important role in the development of necrotic lesions, but their role has not been thoroughly investigated [29].

**Figure-1:**
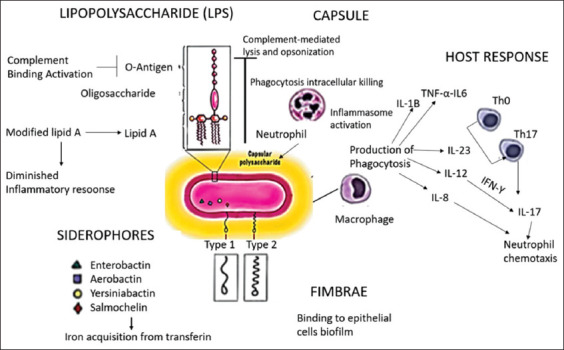
The scheme of host innate immune response and virulence of *Klebsiella pneumonia* [30].

The polysaccharide capsule prevents phagocytosis while also inhibiting complement-mediated lysis and opsonization. Complete LPS will elicit a strong seditious response, aiding in the transfer of C1q to bacteria and igniting the complement pathway. Furthermore, certain *Klebsiella* strains can modify LPS so that it cannot be used by vulnerable cells, whereas others can use the capsule to help the toll-like receptor detect LPS (TLR4). *Klebsiella pneumoniae* also has fimbriae types 1 and 3, which help with adhesion to biotic and abiotic shells, as well as epithelial cell irruption and biofilm conformation. This process also synthesizes siderophores such as aerobactin, enterobactin, salmochelin, and yersiniabactin, to gain iron from the host. Furthermore, through the process of phagocytosis and the production of vulnerable mediators similar to cytokines and chemokines, macrophages play an important role in the ingrain susceptible response. Interleukin (IL)-23 plays an important role in this process by inducing the product of IL-17 and IL-8, which promotes neutrophil reclamation. IL-12 stimulates the expression of IL-17 through IFN-. Another type of cytokine is IL-1, which is produced by activating the seditious pathway for nucleotide oligomerization domain (NOD)-like receptors similar to the pyrin receptor (NLRP3), as well as other pro-inflammatory cytokines such as TNF- and IL-6 (Figure-1) [30].

The LPS is an important element of the external membrane of *Klebsiella*, which is also known as an endotoxin conforming to three corridors antigen O, core oligosaccharides, and lipid A. The genes needed for their conformation are located in the *wb*, *waa*, and *lpx* gene groups. Lipopolysaccharide has a vital part in the acridity of these bacteria, where *K. pneumoniae* can alter lipid A, leading to the inactivation of the seditious response [31]. Furthermore, lipid A blocks the bactericidal effect of antimicrobial peptides. Lipopolysaccharide is the main avenue of defense against complement, wherein strains with full-length O antigen or smooth LPS are resistant to complement-intermediated payoff. In contrast, strains with abbreviated or absent O-chain or crude LPS are sensitive to complement-intermediated payoff indeed in the present capsule [30]. Moreover, it is believed that the part of O antigen is in baffling the attachment of C1q to bacteria, which blocks consecutive stimulants from completing the common pathway by fixing C3b in addition to the bacterial external subcaste and, therefore, frustrated bacterial lysis by the complex attack complement membrane [1].

Lipopolysaccharides are formed from lipid A, oligosaccharide synopsis, O antigens and are known as endotoxins based on all Gram-negative pathogens, including cKP and hvKP. At present, it is not explicit whether the resulting LPS hvKP strain has an individual role in hypervirulence. The outermost subunit based on LPS, O antigen is the primary constituent faced by the innate immune system and protects bacteria against complement-mediated inflammation. In particular, the O antigens bind to the complementary constituent C3b, which involves pore arrangement before mediating the drilling of pathogenic tissue. The number of O serotypes is estimated to be eight, and O1 antigen is the most common among clinical strains of *K. pneumoniae* [32].

During infection, *K. pneumoniae* overcomes mechanical and chemical barriers as well as cellular and humoral host defenses. Furthermore, once inside the host, the host organism attacks the vulnerable cell, which is linked to pattern recognition. Following recognition, the receptor activates the product of central vulnerable intercessors, and the ingrain vulnerable response participates in the monocyte/macrophage system. Furthermore, this system is capable of phagocytosis and regulates the vulnerable response to cytokines and product chemokines. Neutrophils are immune cells that are the first to respond to an infection. Interleukin-8 and IL-23, which both play a role in converting a granulopoietic response, are important cytokine proteins in this phase [1]. Interleukin-12 stimulates the expression of IL-17 by producing interferon-gamma. The product of IL-1 participates in the vulnerable response through activation of the pyrine-containing NOD receptor sphere (NLRP3) in the seditious pathway, as do the products of other pro-inflammatory cytokines such as TNF- and IL-6 [33].

Capsule polysaccharides, LPSs, fimbriae, and siderophores are well-studied virulence factors [1]. Type 1 and type 3 fimbriae of *K. pneumoniae* are equipped with adhesins and are useful for epithelial attachment and cell impunity, as well as abiotic shells. Seventy-eight different capsule serotypes (K1 to K78) have been linked to *K. pneumoniae* based on the structure of the CPs. The hvKp strains are represented by serotypes K1 and K2. The high acridity position of the hvKp strain is due to an excess of capsular material (hypermucoviscos phenotype). Although, the capsule is important in protecting *K. pneumoniae* from the host’s vulnerable response. Furthermore, certain *K. pneumoniae* strains can convert LPS to situations that are not recognized by host cells, while others can use the capsule to conceal LPS from detection by TLR4 [23, 30]. The ability of *K. pneumoniae* to take iron from the host explains their growth and replication, which has been explained by iron motes or siderophores. The hvKP strain can also produce significantly less iron accession and is a more biologically active strain than other non-virulent *K. pneumoniae* strains. Furthermore, no natural marker has yet been identified that can distinguish hvKp from other *K. pneumoniae* strains. Furthermore, MDR hvKP can be caused by two distinct mechanisms. The hvKP strain was able to accept antibacterial agent horizontal gene transfer resistance genes or plasmids, it temporarily became MDR hvKP type I [34]. Multidrug resistance hvKP strains can also be derived from pathogenic plasmids such as pLVPK. Multidrug resistance hvKP type II is the most common MDR strain of *K. pneumoniae*. A recent study in China revealed fatalities. Furthermore, KPC-producing ST11 strains induced the acquisition of pLVPK-like pathogenic plasmid [35]. *Klebsiella pneumoniae* can form microbial cells that irreversibly cleave to the cell’s surface or within the cell to polymeric extracellular matrix substances. Fimbria type 1 and fimbria type 3 are polysaccharide capsules that contribute to the biofilm formation process and are acridity factors. The process starts with the formation of a biofilm by fimbria type 3, which is made up of mrkA protein subunits. Furthermore, mrkD is another element located at the tip of the fimbria that functions as a complement to give tenacious parcels and also determines the fimbria’s listed capacity [36].

In addition, despite the inherent resistance to ampicillin, hvKp strains are generally susceptible to various antibiotics, including cephalosporins and carbapenems. However, MDR and highly toxic strain (MDRhv) have recently emerged, mainly due to plasmid-mediated horizontal transmission of pathogens or resistance [37, 38]. Further, for ambiguous variant potential pathogens, mice, but not *Galleria melonella* is employed. *Galleria melonella* mortality test combined with the string test yields an accurate phenotype, and the test proves to improve clinical identification [39, 40].

*Klebsiella pneumoniae* forms a biofilm and is multidrug-resistant. Fimbriae influence adhesion stability, while CP influences cell-to-cell communication and biofilm structure. The bedded cell must be capable of carrying out rapid-fire and expansive changes in gene expression for the production process of biofilm conformation and variability of stimulants from the terrain. *Klebsiella pneumoniae* cells in the biofilm are only partially protected against vulnerable defenses. The matrix prevents antibacterial antibodies and peptides from reaching the bacteria, as well as reducing or suppressing the effectiveness of complement and phagocytosis [41]. The formation of *K. pneumoniae* biofilms on solid shells was characterized by cell adherence, microcolony conformation, and eventually the dissolution of free-living cells. Type 3 fimbria and CP are important corridors in the process of forming bacterial face structures, which directly and laboriously have the capability of suppressing seditious responses and changing the vulnerable system in habitual infections, to determine resistance, the most important factor being the growth status of bacteria. The core part of the biophilic structure to which bacteria adapt to starvation and low oxygen environments causes bacterial growth to slow, reducing the efficacy of antibiotics that directly target metabolically active and dividing cells. In *K. pneumoniae*, apparent quorum detectors and autoinducers have been described [20].

## The Emergence of Multidrug-resistant Food

The medium of exchange between antibiotic resistance and acridity in *K. pneumoniae* is unknown. *Klebsiella pneumoniae* is frequently associated with nosocomial and salutary infections; this case has also been reported as a possible vector of transmission [42]. *Klebsiella pneumoniae* is no longer found in raw meat, raw vegetables, fruit authorities, or ready-to-eat (RTE) foods. Numerous studies on *K. pneumoniae* in food have been conducted. Antibiotic resistance is of particular concern, with foodborne *K. pneumoniae* being resistant to three or more classes of antibiotics (MDR) [5]. Joint and inter-agency sweats are required to address the issue of antibiotic-resistant bacteria in the food supply chain for public health.

*K. pneumoniae* is found in the normal foliage of animals and humans, food impurity may not be a threat to public health. The discovery of *K. pneumoniae* in food can be related to the hygienic running of food, similar to undercooking or post-cooking impurity, especially for RTE foods. Furthermore, *K. pneumoniae* has also been described to be dominant in husbandry, as *K. pneumoniae* has been shown in previous studies to increase yields under agrarian conditions [43]. In addition, numerous factors, such as the presence of raw vegetables, contribute to the presence of *K. pneumoniae* in food; the addition of organic diseases contributes to the presence of *K. pneumoniae* in food. The discovery of *K. pneumoniae* in raw foods in original requests and supermarkets emphasizes the importance of fresh food safety measures. Moreover, the cross-contamination with bacteria from the raw yield, meat authorities, or other polluted products, or from food instructors with poor particular hygiene, can contaminate cooked RTE foods, even if cooked or packaged safely [44].

*Klebsiella pneumoniae* can be spread through person-to-person contact during the food medication process. As a result, the general public and food consumers should be concerned, because they play a role in the spread (or control) of complaint-causing bacteria. The factors of genetic origin and geographical conditions are often risk factors in this situation [45]. MDR distribution follows a similar geographic pattern and is mostly found in Asian countries. However, it can be found all over the world and has been documented [38, 46].

According to the World Health Organization, food is an implicit means of transmission of antimicrobial-resistant bacteria to humans, and consumption of food containing antimicrobial-resistant bacteria has resulted in antimicrobial resistance. These are global antimicrobial resistance measures, resistant infections, acridity characteristics, and antibiotic resistance biographies required to assess the public health threat posed by foodborne *K. pneumoniae*. According to reports, the most concerning health issue is antimicrobial resistance in *K. pneumoniae* [47, 48]. Foodborne pandemics have occurred in recent decades, reminding us of the importance of enforcing high hygiene standards as well as preventive measures and forestalment programs aimed at food safety for the community, which applies to food products aseptic. Moreover, approaches have also been taken in agriculture and all food production sectors to improve hygiene and reduce natural hazards. The differences in food composition and food processing can all play a role in the emergence of foodborne pathogens. Many cases of foodborne complaints have been reported due to factors such as changes in beneficial habits, increased cross-country travel, changes in food product processing and distribution processes, pathogen adaptation to new environments, antimicrobial resistance by microorganisms, advances in pathogen discovery, sanitation, and vector control measures, poor public health services, and consumer information [6].

An infectious disease epidemic necessitates three critical components: The source, the pathway, and the vulnerable population. In terms of habitat relevance, *K. pneumoniae* has been found in humans, animals, sewage, contaminated water samples, and soil. The origin of hvKP, on the other hand, is unknown [49]. Foods similar to dairy products can be a source of transmission of Enterobacteriaceae microorganisms that exhibit MDR to antibiotics and other acridity factors similar to biofilm products, as well as the conflation of proteolytic and lipolytic enzymes that are responsible for corruption processes in food products. Good hygiene conditions during processing and manufacturing, as well as storage and distribution processes can reduce or eliminate the presence of these microorganisms [50]. The emergence of antimicrobial resistance in *K. pneumoniae* is a major concern in life-saving drugs around the world. *Klebsiella pneumoniae* multidrug-resistant strains have been identified and isolated from various samples [51].

Food is one of the factors that contribute to the occurrence of antibiotic-resistant bacteria and their genes in the mortal digestive tract. The prevalence of foodborne illnesses caused by *K. pneumoniae* has recently increased. There is currently little information available on the characteristics of *K. pneumoniae* that has been isolated from food. Certain food orders may influence the diversity of gut antibiotic resistance genes [52, 53]. Table-1 shows data on the factors that contribute to the toxicity of various types of food [54–56]. Furthermore, these bacteria can transfer the genes that determine antibiotic resistance to other types of pathogenic bacteria. As a result, surveillance and monitoring of antimicrobial-resistant bacteria in food are critical for enforcing targeted control strategies and selecting effective treatment options [57–59].

**Table-1:** Factors contributing to toxicity from different kinds of food.

Food	virulence Factor	Antibiotic resistance/Resistance gene variant	Reference
Cooked meat	-	*bla*-B-10	[54]
Raw meat	-	*bla* CTX-M-15, *bla* SHV28, *bla* Tem-1B	
Noodles	K54, *wca* G	-	
Beverage	K2	-	
Pork dishes	*wca* G	-	
Chili’s	K, *wca* G	AMP-TE-AK-C-SXT	
Lettuce	K1, *wca* G	-	
Pork liver	K2	AMP-C-CIP-TE-SXT	
Porridge	*wca* G	-	
Black tiger shrimp	-	*bla* CTX, *bla* SHV	
Cooked and raw meat	-	*blaOKP*-B-10	[55]
Uncooked vegetables	-	*aac*(3)-*IIc*	[56]

## Conclusion

The emergence of MDR strains and the rise of *K. pneumoniae* have compelled scientists and researchers to seek out and define newer antibacterial treatments. There is a clear need to define and engage the public to increase surveillance of *K. pneumoniae* in food, as well as to improve our understanding of the epidemiological and public health implications of this foodborne pathogen. As a result, it is critical to recognize that *K. pneumoniae* is a major disease pathogen that can be transmitted through the food chain, and this must be done immediately.

## Authors’ Contributions

KHPR: Conceived the idea and drafted and revised the manuscript. MHE and FAR: Reviewed the manuscript. KHPR, AW, and ARK: Literature searches and edited and reviewed the manuscript. All authors have read and approved the final manuscript.
